# Validation of Reference Genes for RT-qPCR Studies of Gene Expression in Preharvest and Postharvest Longan Fruits under Different Experimental Conditions

**DOI:** 10.3389/fpls.2016.00780

**Published:** 2016-06-03

**Authors:** Jianyang Wu, Hongna Zhang, Liqin Liu, Weicai Li, Yongzan Wei, Shengyou Shi

**Affiliations:** ^1^Department of Biochemistry, Basic Education College of Lingnan Normal UniversityZhanjiang, China; ^2^Key Laboratory of Tropical Fruit Biology, South Subtropical Crops Research Institute, Chinese Academy of Tropical Agricultural Sciences, Ministry of AgricultureZhanjiang, China

**Keywords:** Longan (*Dimocarpus longan* Lour.), preharvest and postharvest fruits, gene expression, reference genes, RT-qPCR, normalization, *ACO* gene

## Abstract

Reverse transcription quantitative PCR (RT-qPCR) as the accurate and sensitive method is use for gene expression analysis, but the veracity and reliability result depends on whether select appropriate reference gene or not. To date, several reliable reference gene validations have been reported in fruits trees, but none have been done on preharvest and postharvest longan fruits. In this study, 12 candidate reference genes, namely, *CYP, RPL, GAPDH, TUA, TUB, Fe-SOD, Mn-SOD, Cu/Zn-SOD, 18SrRNA, Actin, Histone H3*, and *EF-1a*, were selected. Expression stability of these genes in 150 longan samples was evaluated and analyzed using geNorm and NormFinder algorithms. Preharvest samples consisted of seven experimental sets, including different developmental stages, organs, hormone stimuli (NAA, 2,4-D, and ethephon) and abiotic stresses (bagging and girdling with defoliation). Postharvest samples consisted of different temperature treatments (4 and 22°C) and varieties. Our findings indicate that appropriate reference gene(s) should be picked for each experimental condition. Our data further showed that the commonly used reference gene *Actin* does not exhibit stable expression across experimental conditions in longan. Expression levels of the *DlACO* gene, which is a key gene involved in regulating fruit abscission under girdling with defoliation treatment, was evaluated to validate our findings. In conclusion, our data provide a useful framework for choice of suitable reference genes across different experimental conditions for RT-qPCR analysis of preharvest and postharvest longan fruits.

## Introduction

Reverse transcription quantitative PCR (RT-qPCR) as the sensitive and accurate method for gene expression analysis is employed in a wide range of applications (Bustin, 2002). However, the accuracy of RT-qPCR is affected by many factors, including differences in RNA sample quality and extraction method, and efficiencies of reverse transcription and PCR (Die et al., 2010; Schmidt and Delaney, 2010). Use of appropriate reference genes could help eliminate the variability introduced by differences across samples and ensure the veracity and reliability of RT-qPCR results.

A suitable reference gene should possess invariant expression across all samples and under different experimental conditions. Housekeeping genes are often selected as ideal reference genes because they are thought to exhibit such properties. Nevertheless, a few recent reports have indicated that even the widely used reference genes are not invariant under different experimental conditions (Nicot et al., 2005; Marino et al., 2008). GAPDH and Actin are reference genes that exhibit different expression levels in different plants, tissues and experimental conditions (Yperman et al., 2004; Barber et al., 2005). Gutierrez et al. (2008) also found that the gene expression of *TUB, Actin, UBQ*, and *EF* were varies across all samples. If the reference gene used for normalization in RT-qPCR was variant, it will led to inaccuracy conclusion (Czechowski et al., 2005; Gutierrez et al., 2008; Artico et al., 2010). Hence, reference genes must be systematically evaluated to ensure their stability of expression in each experimental system to obtain accurate and reliable results (Lee et al., 2002; Hong et al., 2008). Moreover, the optimal number of reference genes should be determined, as most gene expression analyses are expected to require multiple reference genes (Vandesompele et al., 2002).

To date, several reliable reference gene validations have been reported in fruits such as litchi (Zhong et al., 2011), banana (Chen et al., 2011), papaya (Zhu et al., 2012), apple (Tatiane et al., 2015), citrus (Mafra et al., 2012), berry (Reid et al., 2006), peach (Tong et al., 2009), pear (Wu et al., 2012; Xu et al., 2015), plum (Kim et al., 2015), melon (Carmen et al., 2015), watermelon (Kong et al., 2014), strawberry (Clancy et al., 2013), olive (Tjasa et al., 2013), and grapevine (Mauricio et al., 2013). Such reference gene validations have also been reported for other species such as *Lilium regale* (Liu et al., 2016), celery (Li et al., 2016), Lycoris aurea (Ma et al., 2016), tall fescue (Yang et al., 2015), moss (Li et al., 2015a), carrot leaves (Tian et al., 2015), *Oxytropis ochrocephala* Bunge (Zhuang et al., 2015), cotton (Tu et al., 2007; Artico et al., 2010), tobacco (Schmidt and Delaney, 2010), *Petunia* (Mallona et al., 2010), *Arabidopsis* (Czechowski et al., 2005), potato (Nicot et al., 2005), *Lolium temulentum* (Dombrowski and Martin, 2009), rice (Jain et al., 2006), zucchini (Obrero et al., 2011), and cucumber (Wan et al., 2010). However, there was still no report about selecting appropriate reference genes for RT-qPCR analysis in preharvest and postharvest longan fruits. Although Lin and Lai (2010, 2013) identified reference genes for longan, their results are only applicable for somatic embryogenesis of longan.

Longan (*Dimocarpus longan* Lour.), which belongs to the family of *Sapindaceae*, is an important tropical/subtropical fruit species. The main countries producing this fruit include China, Thailand and Vietnam. Several areas in India, Laos, Philippines, Myanmar, Malaysia and Indonesia also cultivate longan (Shi et al., 2015). Longan fruits are preferably eaten fresh, and they have a delicate and sweet-tasting flesh. This fruit can also be processed to make dried pulp, canned fruit, jam, drinks and wine. In China, longan fruit is mostly marketed locally but has been increasingly imported in recent years (Jiang et al., 2002).

Several preharvest and postharvest problems significantly reduce the yield and commercial value of longan (Jiang et al., 2002). These include fruit abscission during growth and development in preharvest, and fruit deterioration and pericarp browning, reducing the storage potential and marketability of longan, in postharvest periods. The cause of these problems may be at the physicochemical, biochemical and molecular levels (Lai et al., 2001; Jiang et al., 2002). RT-qPCR is a useful method to find the key genes relate to that processes, but there was still no report about selecting appropriate reference genes for RT-qPCR analysis in longan fruits. Although a few gene expression patterns have been reported in longan fruit using different references genes such as *Actin* (Zhao et al., 2011; Chen et al., 2012; You et al., 2014; Shuai et al., 2016), *EF-1a* (Shuai et al., 2016), and *GAPDH* (Shuai et al., 2015), stability of these reference genes has not been verified to date. Therefore, identification of appropriate references genes for RT-qPCR analyses in longan fruit is urgently needed.

In the present study, 12 reference genes, including cyclophilin (*CYP*), ribosomal protein L (*RPL*), glyceraldehyde-3-phosphate dehydrogenase (*GAPDH*), alpha-tubulin (*TUA*), beta-tubulin (*TUB*), copper/zinc-superoxide dismutase (*Cu/Zn-SOD*), manganese superoxide dismutase (*Mn-SOD*), iron-superoxide dismutase (*Fe-SOD*), 18S ribosomal RNA (*18SrRNA*), *Actin, Histone H3* and elongation factor 1-alpha (*EF-1a*) were selected based on evidence of stable expression in previous studies (Lin and Lai, 2010; Chen et al., 2011; Zhong et al., 2011; Zhu et al., 2012).

geNorm and NormFinder were useful programs to identify appropriate reference genes for RT-qPCR analysis, especially using together (Chen et al., 2011; Zhong et al., 2011). Base on the previous reports, this study selected the two programs to rank the stability of the candidate reference genes. Finally, identifying the suitable reference genes for RT-qPCR normalization in preharvest and postharvest longan fruits. The preharvest experiment consisted of seven sample sets, including different developmental stages, organs, hormone stimuli (NAA, 2,4-D, and ethephon) and abiotic stresses (bagging and girdling with defoliation). The postharvest experiment consisted of different temperature treatments (4 and 22°C) and longan varieties.

In addition, for verifying our results, the expression levels of 1-aminocyclopropane-1-carboxylate oxidase (*ACO*) gene under girdling with defoliation treatment was normalized to the most stable and unstable genes. Our findings provide important guidelines for selecting suitable reference genes under different experimental conditions in preharvest and postharvest longan fruits.

## Materials and methods

### Plant materials and treatments

Longan trees were selected in the South Subtropical Crops Research Institute, Chinese Academy of Tropical Agricultural Sciences, Zhanjiang, China (2013). There were a total of 150 samples in this study. Pericarp and aril samples of six varieties: “Shixia,” “Chuliang,” “Linglong,” “Gushan,” “Caopushi,” and “Benzhan” were obtained at 110 days after anthesis (DAA). Pericarp and aril samples at six different developmental stages of “Shixia” (62, 76, 83, 89, 103, and 110 DAA) were collected. The tender root, shoot, young leaf, flower, fruitlets, and seed of “Shixia” were also sampled. The fruitlets and seeds were sampled at 40 and 110 DAA, respectively.

Three 10-year-old “Shixia” trees (2013) were used for treatment, and each tree was used as a biological replicate. The treatment was carried out at 90 DAA. Fifty similar size of the fruit-bearing shoots from each tree were tagged. 10 shoots were treated with 100 mg/L NAA. Meanwhile, 10 shoots were sprayed with 20 and 250 mg/L 2,4-D and ethephon, respectively, 10 shoots were bagged with adhesive-bonded fabric bags and the remaining 10 shoots were control that treated with distilled water. 10 fruits randomly collected from 10 shoots at 0, 2, and 3 d after treatment, and the pericarp was collected as sample.

For temperature samples, the fresh postharvest longan fruits (110 DAA in 2013) were put into unsealed plastic bags and then treated with 4°C (cold temperature) and 22°C (room temperature), respectively. Aril was collected at 2 and 3 d after treatment.

Three 10-year-old “Shixia” trees were selected for girdling plus defoliation treatment, and each tree was a biological replicate. The treatment was performed at 40 DAA. Twenty fruit-bearing shoots were tagged with a uniform diameter from each tree. 10 shoots were girdled at a width of 0.5 cm and defoliated all leaves above the girdle, and the remaining untreated 10 shoots were used as control. The fruitlets and fruit abscission zones of 10 fruits randomly harvested from 10 fruit-bearing shoots were sampled at 4 and 5 d after treatment. Fruit abscission zone samples were collected by cutting ~1 mm piece at each side of the abscission fracture plane.

Table 1 summarizes the data of all sample sets mentioned above. All tissues were immediately frozen in liquid nitrogen after separation and stored at −80°C for future use.

**Table 1:** **Sample sets considered in the present study**.

**Experimental sample sets**	**Tissue type**	**Sampling dates**	**Cultivars**	**Biological replicates**	**Total number of samples**
Different varieties	Pericarp, aril	110 DAA	“Shixia,” “Chuliang,” “Linglong,” “Gushan,” “Caopushi,” and “Benzhan”	3	36
Different developmental stages	Pericarp, aril	62, 76, 83, 89, 103, 110 DAA	“Shixia”	3	36
Different organs	Root, shoot, leaf, flower, fruitlets seed	40, 110 DAA	“Shixia”	3	18
NAA	Pericarp	0, 2, 3 DAT	“Shixia”	3	9
2,4-D	Pericarp	0, 2, 3 DAT	“Shixia”	3	9
Ethephon	Pericarp	0, 2, 3 DAT	“Shixia”	3	9
Bagging	Pericarp	0, 2, 3 DAT	“Shixia”	3	9
Temperature (4°C, 22°C)	Aril	2, 3 DAT	“Shixia”	3	12
Girdling with defoliation	Fruitlets, fruit abscission zone	4, 5 DAT	“Shixia”	3	12

DAA, days after anthesis; DAT, days after treatment. Total number of samples: 150.

### RNA isolation, quality control, and cDNA synthesis

According to the protocol of Wu et al. (2011), total RNA was extracted from the frozen samples. DNase I (TaKaRa, Otsu, Japan) and RNAse-free columns (Huayueyang, Beijing, China) were used to remove potentially contaminating DNA and purify total RNA, respectively. RNA integrity was confirmed using Agilent 2100 Bioanalyzer (Agilent Technologies, Santa Clara, CA, USA). For reverse transcription, 2 μg of total RNA and oligo-dT primers were used with the M-MLV cDNA synthesis kit following the manufacturer's instructions (Promega).

### Selection of candidate reference genes for preharvest and postharvest longan fruits

Based on previous studies, 12 reference genes were selected to compare the stability of their expression across samples. *CYP* and *RPL* were selected from our transcriptome database (Zhang et al., 2016; http://www.ncbi.nlm.nih.gov/sra/SRX1395356/). *GAPDH* (GeneBank number FJ694012), *TUA* (GeneBank number FJ479617), *TUB* (GeneBank number KC921220), *Fe-SOD* (GeneBank number EU330204), *Mn-SOD* (GeneBank number EU563945), and *Cu/Zn-SOD* (GeneBank number EU157910) were obtained from NCBI. *18SrRNA, Actin, Histone H3* and *EF-1a* were picked based on the report by Lin and Lai (2010).

### Design and validation of RT-qPCR primers

Primer pairs of *CYP, RPL, GAPDH, TUA, TUB, Fe-SOD, Mn-SOD*, and *Cu/Zn-SOD* were designed using Primer Premier 5.0 software, while primer pairs of *18SrRNA, Actin, Histone H3*, and *EF-1a* were selected based on the report by Lin and Lai (2010). All primers were obtained from a commercial supplier (Sangon, Guangzhou, China). Prior to RT-qPCR, each primer pair was tested via melting curve, the good melting curve should meet the requirement that there was a single peak in the template and no amplicon was observed in no template control (NTC). The size specificity of the amplicon were confirmed by using 2.0% agarose gel electrophoresis. In orded to verify the cDNA sequence of the PCR products the target amplicons were sequenced. Standard curve was used to calculate the PCR efficiency for each gene. Table 2 shows the selected candidate reference genes, primers, and amplicon characteristics.

**Table 2:** **Selected candidate reference genes, primers, and amplicon characteristics**.

**Gene**	**Primer sequences (forward/reverse)**	**Amplicon length (bp)**	**Tm (°C)**	**Amplification efficiency**
*CYP*	AAGGAGATGGAGAAGGTTGGTT	213	83.37	2.072
	CCCTTTCTGACTTTGGGTAGACATA			
*RPL*	TCAGACAGTGATTACACCGAGTTC	246	80.87	1.914
	GCCAGTAGAGACAAAAAGGCAAGA			
*GAPDH*	GTCGCTTGGCTGCCTATAATCT	204	81.79	1.962
	AGACCGTTGACTGAACCATCC			
*TUA*	GATTATGAGGAAGTTGGGGCTG	175	81.09	1.986
	AAGCAACCTAGCACATAGTGAAAGT			
*TUB*	AGATGTTCCGTCGTGTGAGTGA	86	83.43	1.977
	CCTCCTCATATTCATCCTCATCAG			
*Fe-SOD*	AAGAGGAGAAAGAGCAAGAGTCAGA	114	80.80	1.917
	CCGATACAACAAACCCTGAAATG			
*Mn-SOD*	ACTACCTACAGTACAAGAATGTCAGACC	184	81.44	1.949
	GGGCTCCTCATCCTATATCGTT			
*Cu/Zn-SOD*	TTGTAGGAAGGGCTGTCGTTG	207	82.46	1.992
	TCTCGTCAAGTCACTCTCAAGCAT			
*18SrRNA*	CCTGAGAAACGGCTACCACAT	171	83.93	1.931
	CACCAGACTTGCCCTCCA			
*Actin*	TGCTATCCTTCGGTTGGACC	93	81.77	1.984
	CGGACGATTTCCCGTTCAG			
*Histone H3*	ATCCGCAAGTACCAGAAGAGCA	155	85.71	2.002
	CCCACCAAGTAAGCCTCAG			
*EF-1a*	GATGATTCCCACCAAGCCCAT	129	84.57	2.072
	GGGTCCTTCT TCTCAACACTCT			

### RT-qPCR conditions

RT-qPCR was conducted on a LightCycler480 II System (Roche, Switzerland) using DyNAmo Flash SYBR Green q-PCR kit (Thermo, USA). Each reaction mix contained 1 and 5 μL of diluted cDNAs and SYBR Green PCR Master Mix (Thermo, USA), respectively, and 250 nM of each primer to a final volume of 10 μL. The amplification conditions were as follows: 50°C for 2 min; 95°C for 10 min; 40 cycles at 95°C for 15 s; 55°C for 30 s; and 72°C for 30 s in 384-well optical reaction plates (Roche, Switzerland). The dissociation curve was analyzed at 60–95°C over 40 cycles to determine primer specificity. Each assay included three technical and biological replicates.

### Data analysis

The stability of the 12 selected reference genes were ranked by geNorm and NormFinder algorithms, and the gene expression levels were determined using crossing point (Cp) values. The raw Cp values should transform to relative quantities called the Q values, when the data input into the two algorithms. The Q values were calculated using the equation: Q = 2^(minCp−sampleCp)^. After the geNorm algorithm was applied, the stability value (M) for each gene was generated, and pairwise variation (V) between genes was obtained. The M and V values indicate the most appropriate reference genes and the optimal number of reference genes, respectively (Vandesompele et al., 2002). The NormFinder algorithm calculates the stability for each gene, with lower stability value indicating more stable reference genes (Andersen et al., 2004).

### Normalization of *DlACO*

The *ACO* gene converting ACC into ethylene. *ACO* gene expression is positively correlated with ethylene production rates and upregulated during fruit abscission (Li and Yuan, 2008a,b; Li et al., 2015b). One *DlACO* gene (GeneBank number GU123929.1) was used to verify the results of reference gene stability analysis in RT-qPCR. Gene expression level of *DlACO* in the fruit abscission zone was quantified during girdling with defoliation using one or two most suitable reference genes and the most unsuitable gene that determined by geNorm. The primer pairs (forward: 5′-CAACTTGAGGTTATCACAAATGGG -3′ and reverse: 5′-ACAATGCTGGAGCTGGGTAGA -3′) for *DlACO* were also confirmed with the same methods described above.

## Results

### Verification of amplicons, primer specificity, and PCR amplification efficiency

The agarose gel electrophoresis showed there were only one amplicon with the designed size after PCR amplified in all candidate reference genes, and the same results were observed in melting curve. The melting curve showed a single peak in the template and no amplicon was observed in NTC for each selected reference gene (Figure S1). Furthermore, sequence analysis showed that all of the sequenced amplified fragments were matched to the sequences used for primer design. The PCR efficiency for the 12 reference genes between 1.914 (*RPL*) and 2.072 (*CYP*; Table 2).

### Cq values of candidate reference genes in preharvest and postharvest longan fruits

**Figure 1:**
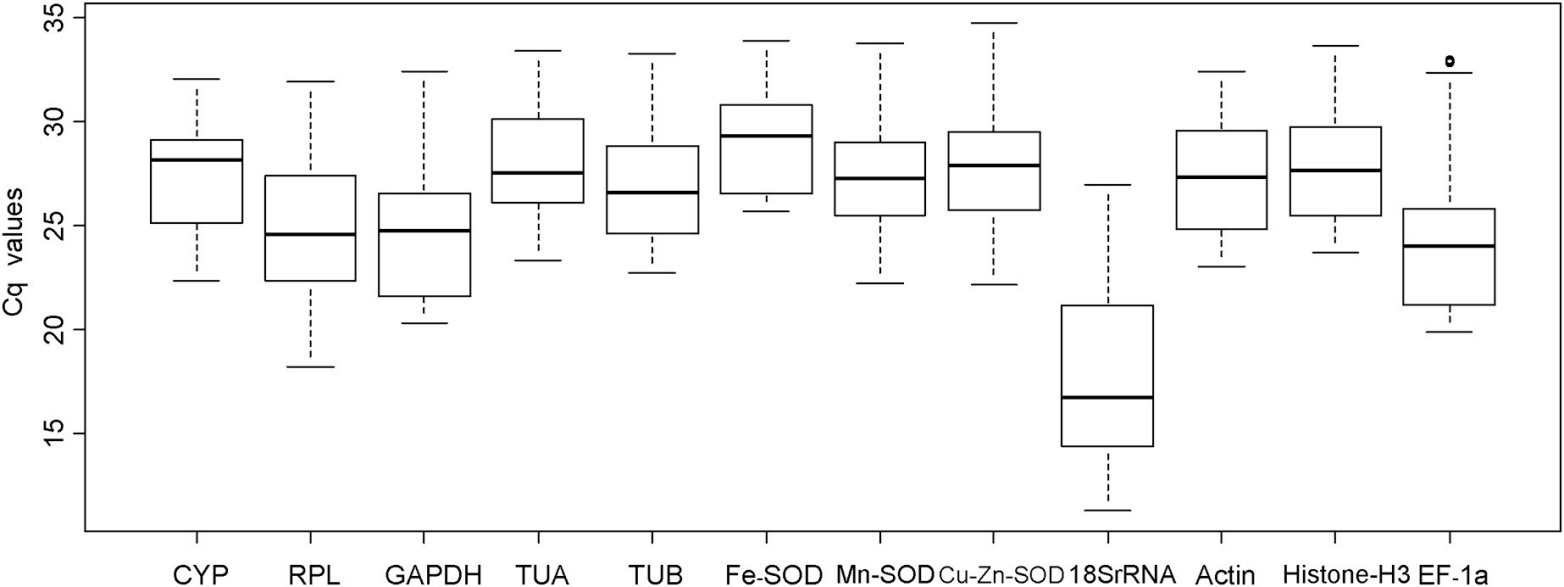
**Cq values of candidate reference genes in all longan samples**. Lines across boxes indicate median values. Boxes indicate 25/75 percentiles. Whisker caps indicate the minimum and maximum values. Circles represent outliers.

All the Cq values of the reference genes are shown in Table S1. The Cq values of the 12 reference genes were variable (Figure 1), ranging between 11.25 (*18SrRNA*) and 34.72 (*Cu/Zn-SOD*), while the mean Cq values ranged between 17.43 (*18SrRNA*) and 29.02 (*Fe-SOD*; Table S2). Moreover, *18SrRNA* levels were the most variable (15.73 Cq, the maximun and minimum values were 26.98 and 11.25, respectively) while *Fe-SOD* was the least variable (8.21 Cq, the maximun and minimum values were 33.88 and 25.67, respectively). Since Cq values are negatively correlated to expression levels, our data indicate that *18SrRNA* had high expression while *Fe-SOD* had low expression (Table S2).

### Determination of the optimal number of reference genes in preharvest and postharvest longan fruits

**Figure 2:**
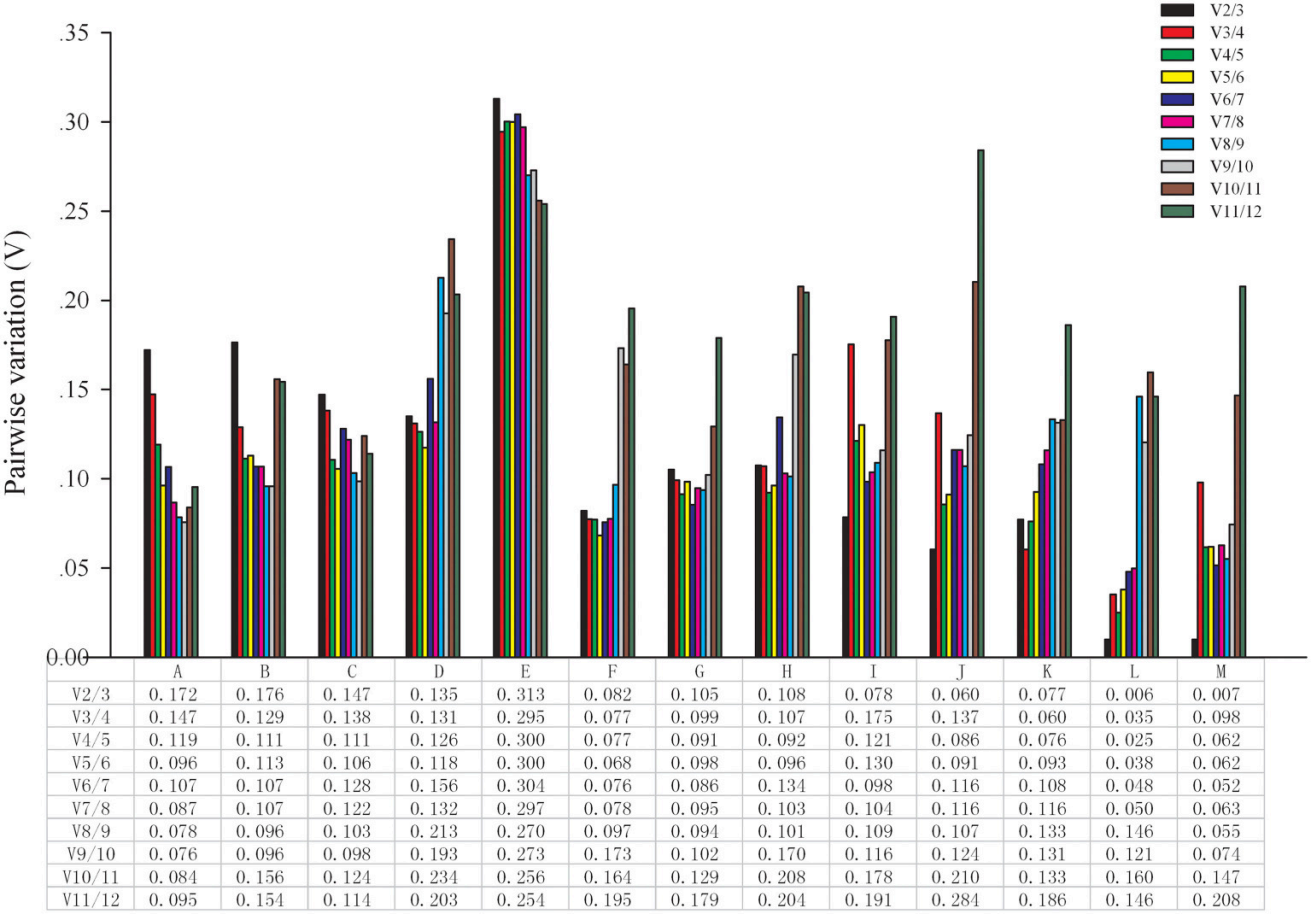
**Determination of the optimal number of reference genes**. Pairwise variation (Vn/n +1) analysis between the normalization factors (NFn and NFn +1) was performed using the geNorm program in all samples. A, different varieties (pericarp); B, different varieties (aril); C, different developmental stages (pericarp); D, different developmental stages (aril); E, different organs; F, NAA stimuli; G, 2,4-D stimuli; H, ethephon stimuli; I, bagging; J, cold temperature treatment (4°C); K, room temperature treatment (22°C); L, girdling with defoliation (fruit abscission zone); M, girdling with defoliation (fruitlets).

The optimal number of reference genes was determined using geNorm. The software can calculate the pairwise variation (V) values of normalization factor by introducing new reference genes, and the most appropriate number is determined according to the ratio Vn/Vn+1. As shown in Figure 2, two reference genes were sufficient for normalizing gene expression at different developmental stages, NAA, 2,4-D, ethephon, bagging, temperature (4 and 22°C) and girdling with defoliation treatments because the V2/3 value was lower than 0.15. Three reference genes were sufficient for RT-qPCR analysis in different varieties because the V2/3 value (0.172 and 0.176) was higher than 0.15, and the V3/4 value (0.147 and 0.129) was lower than 0.15. However, none of the genes selected was appropriate for different organ samples because all Vn/n +1 values were higher than 0.15.

### Expression stability of candidate reference genes in preharvest and postharvest longan fruits

**Figure 3:**
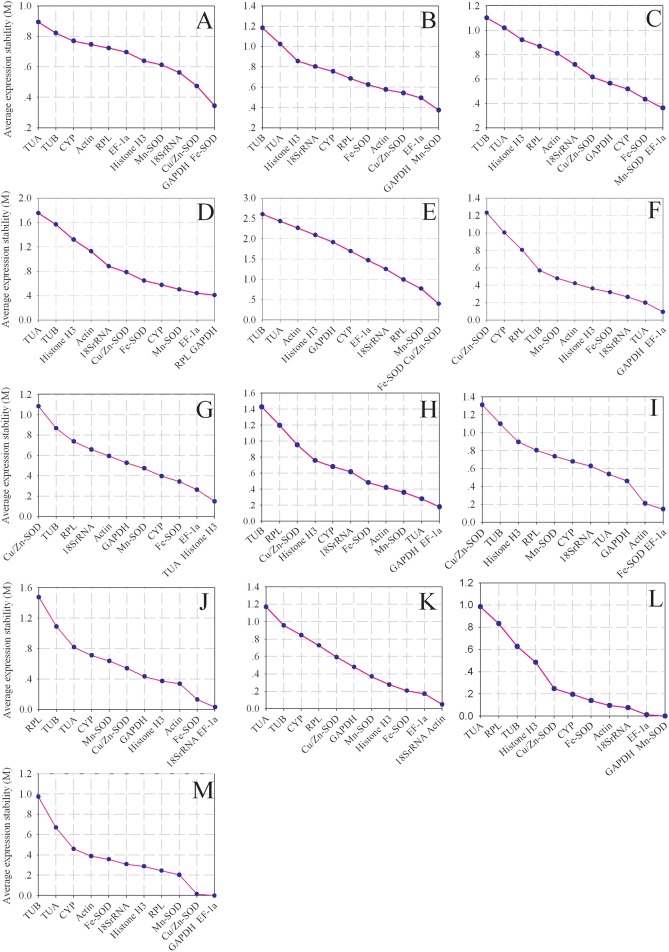
**Average expression stability values (M) of candidate reference genes**. The average M values of the reference genes were measured by stepwise exclusion of the least stable reference genes. A lower M value indicates more stable expression, as analyzed by the geNorm software in longan sample sets under different experimental conditions. **(A)** different varieties (pericarp), **(B)** different varieties (aril), **(C)** different developmental stages (pericarp), **(D)** different developmental stages (aril), **(E)** different organs, **(F)** NAA stimuli, **(G)** 2,4-D stimuli, **(H)** ethephon stimuli, **(I)** bagging, **(J)** cold temperature treatment (4°C), **(K)** room temperature treatment (22°C), **(L)** girdling with defoliation (fruit abscission zone), **(M)** girdling with defoliation (fruitlets).

The results were shown in Figure 3 and summarized in Table S3 by geNorm algorithm. The *GAPDH* and *Fe-SOD* genes were the most suitable candidates for normalization in the pericarp of longan fruit in different varieties with an M value of 0.343 (Figure 3A), whereas *GAPDH* and *Mn-SOD* genes were the most suitable genes in the aril of longan fruit in different varieties with an M value of 0.374 (Figure 3B). For the pericarp or aril of longan fruit samples at different developmental stages, the most stable genes were *Mn-SOD* and *EF-1a*, and *GAPDH* and *RPL* with M values of 0.362 and 0.409, respectively (Figures 3C,D). *Fe-SOD* and *Cu/Zn-SOD* were the most stable genes across different organ samples with an M value of 0.398 (Figure 3E). The *GAPDH* and *EF-1a* genes ranked high in the pericarp of longan fruit under NAA and ethephon conditions, with M values of 0.093 and 0.179, respectively (Figures 3F,H). *TUA* and *Histone H3* genes were the most stable genes in the pericarp of longan fruit under 2,4-D conditions with an M value of 0.147 (Figure 3G). *Fe-SOD* and *EF-1a* were the most appropriate genes with an M value of 0.145 in the bagging condition (Figure 3I). For temperature treatments at 4 and 22°C, the gene pairs with the highest expression stability were *18SrRNA* and *EF-1a*, and *18SrRNA* and *Actin* with M values of 0.031 and 0.050, respectively (Figures 3J,K). For girdling with defoliation treatment, the suitable genes were *GAPDH* and *Mn-SOD* genes with an M value of 0.000 in the fruit abscission zone, and *GAPDH* and *EF-1a* genes with an M value of 0.000 in fruitlets (Figures 3L,M). These results indicate that different reference genes should be selected depending on experimental conditions.

According to the optimal number of reference genes in longan fruit obtained from geNorm analysis, the most suitable combinations for 12 sample sets were *GAPDH* + *Fe-SOD* + *Cu/Zn-SOD* (pericarp of different varieties), *GAPDH* + *Mn-SOD* + *EF-1a* (aril of different varieties), *Mn-SOD* + *EF-1a* (pericarp of different developmental stages), *GAPDH* + *RPL* (aril of different developmental stages), *GAPDH* + *EF-1a* (pericarp of NAA and ethephon treatment), *TUA* + *Histone H3* (pericarp of 2,4-D treatment), *Fe-SOD* + *EF-1a* (pericarp of bagging treatment), *18SrRNA* + *EF-1a* (4°C temperature treatment), *18SrRNA* + *Actin* (22°C temperature treatment), *GAPDH* + *Mn-SOD* (fruit abscission zone of girdling with defoliation treatment), and *GAPDH* + *EF-1a* (fruitlets of girdling with defoliation treatment). However, none of the possible gene combinations was deemed suitable for the different organ samples since all of the Vn/n +1 values were higher than 0.15.

**Figure 4:**
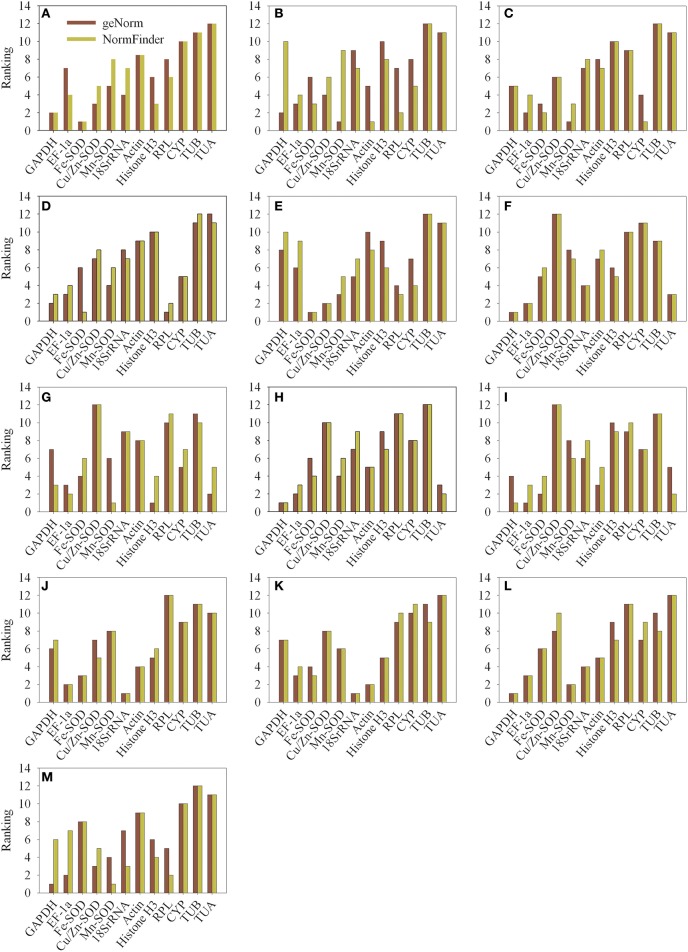
**Comparison of the ranking of the candidate reference genes according to their stability values calculated using geNorm and NormFinder. (A)** different varieties (pericarp), **(B)** different varieties (aril), **(C)** different developmental stages (pericarp), **(D)** different developmental stages (aril), **(E)** different organs, **(F)** NAA stimuli, **(G)** 2,4-D stimuli, **(H)** ethephon stimuli, **(I)** bagging, **(J)** cold temperature treatment (4°C), **(K)** room temperature treatment (22°C), **(L)** girdling with defoliation (fruit abscission zone), **(M)** girdling with defoliation (fruitlets).

The NormFinder program was used to further verify the results obtained from geNorm analysis. The results were shown in Table S4 by NormFinder analysis. Some differences were observed in the results obtained from geNorm and NormFinder analyses. The six most stable reference genes (half of the total) were almost similar, but the ranking orders were different in the results yielded by the two algorithms (Figure 4 and Table S5). Table 3 summarizes the same results got from geNorm and NormFinder programs. In all experimental conditions, the most appropriate gene outcome by NormFinder analysis was in the top six appropriate genes obtain by geNorm analysis. In addition, the two most unstable genes were the same in both geNorm and NormFinder analyses.

**Table 3:** **Consensus of stability ranking of the reference genes estimated by geNorm and NormFinder**.

**Experimental sample sets**	**Six most stable genes by geNorm**	**Most stable genes by NormFinder**	**Two least stable genes by geNorm and NormFinder**
Different varieties (pericarp)	*Fe-SOD, GAPDH, Cu/Zn-SOD, 18SrRNA, Mn-SOD, Histone H3*	*Fe-SOD*	*TUA, TUB*
Different varieties (aril)	*GAPDH, Mn-SOD, EF-1a, Cu/Zn-SOD, Actin, Fe-SOD*	*Actin*	*TUB, TUA*
Different developmental stages (pericarp)	*Mn-SOD, EF-1a, Fe-SOD, CYP, GAPDH, Cu/Zn-SOD*	*CYP*	*TUB, TUA*
Different developmental stages (aril)	*GAPDH, RPL, EF-1a, Mn-SOD, CYP, Fe-SOD*	*Fe-SOD*	*TUB, TUA*
Different organs	*Fe-SOD, Cu/Zn-SOD, Mn-SOD, RPL, 18SrRNA, EF-1a*	*Fe-SOD*	*TUB, TUA*
NAA treatment (pericarp)	*GAPDH, EF-1a, TUA, 18SrRNA, Fe-SOD, Histone H3*	*GAPDH*	*Cu/Zn-SOD, CYP*
2,4-D treatment (pericarp)	*TUA, Histone H3, EF-1a, Fe-SOD, CYP, Mn-SOD*	*Mn-SOD*	*Cu/Zn-SOD, TUB/RPL*
Ethephon treatment (pericarp)	*GAPDH, EF-1a, TUA, Mn-SOD, Actin, Fe-SOD*	*GAPDH*	*TUB, RPL*
Bagging treatment (pericarp)	*Fe-SOD, EF-1a, Actin, GAPDH, TUA, 18SrRNA*	*GAPDH*	*Cu/Zn-SOD, TUB*
Cold temperature treatment (4°C aril)	*18SrRNA, EF-1a, Fe-SOD, Actin, Histone H3, GAPDH*	*18SrRNA*	*RPL, TUB*
Room temperature treatment (22°C aril)	*18SrRNA, Actin, EF-1a, Fe-SOD, Histone H3, Mn-SOD*	*18SrRNA*	*TUA, TUB/ CYP*
Girdling with defoliation (fruit abscission zone)	*GAPDH, Mn-SOD, EF-1a, 18SrRNA, Actin, Fe-SOD*	*GAPDH*	*TUA, RPL*
Girdling with defoliation (fruitlets)	*GAPDH, EF-1a, Cu/Zn-SOD, Mn-SOD, RPL, Histone H3*	*Mn-SOD*	*TUB, TUA*

### Reference gene validation

**Figure 5:**
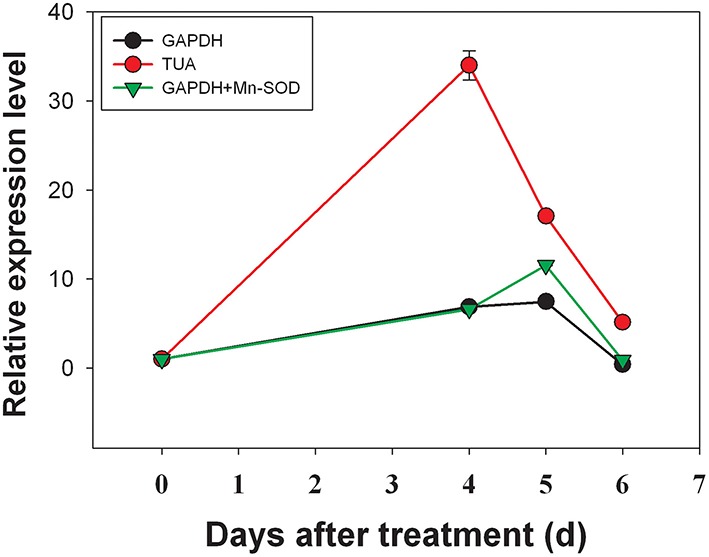
**Relative quantification of *DlACO* expression using validated reference genes for normalization under girdling with defoliation conditions**.

The expression level of *DlACO* under girdling with defoliation treatment was evaluated to validate our findings. The results were shown in Figure 5. The analysis revealed that the expression level of *DlACO* in fruit abscission zone increased progressively after girdling with defoliation treatment. Similar change patterns with minor differences were observed when using *GAPDH* alone or combination of *GAPDH* + *Mn-SOD* as reference gene(s). While, when using *TUA* gene (the least stable) as reference gene resulted in overestimation of *DlACO* expression (Figure 5). The effect was clearly caused by low stability of *TUA* gene expression in the sample and indicated that an appropriate reference gene was urgently needed in RT-qPCR analysis.

## Discussion

RT-qPCR as the accurate and sensitive method for gene expression analysis, but the veracity and reliability result depends on whether select appropriate reference gene or not (Nicot et al., 2005). A suitable reference gene should possess invariant expression across all samples and in different experimental conditions. However, no gene has been found to meet this requirement (Artico et al., 2010). Therefore, it is important to find the appropriate reference gene(s) suitable for RT-qPCR normalization in the tissue or experimental condition of interest (Czechowski et al., 2005; Narsai et al., 2010). As far as we know, this study was the first to systematically select appropriate reference genes for RT-qPCR analysis in preharvest and postharvest longan fruits.

In this study, twelve candidate reference genes that have been validated previously in tropical/subtropical fruit trees were selected. *Actin* and *EF-1a* have been reported previously in somatic embryogenesis of longan (Lin and Lai, 2010), litchi (Zhong et al., 2011), banana (Chen et al., 2011), and papaya (Zhu et al., 2012); *GAPDH* and *TUA* have been validated in litchi (Zhong et al., 2011), banana (Chen et al., 2011), and papaya (Zhu et al., 2012); *CYP* has been studied in banana (Chen et al., 2011) and papaya (Zhu et al., 2012); *18SrRNA* has been used to study somatic embryogenesis of longan (Lin and Lai, 2010) and papaya (Zhu et al., 2012); *TUB* has been reported in somatic embryogenesis of longan (Lin and Lai, 2010) and litchi (Zhong et al., 2011); *Fe-SOD, Mn-SOD, Cn/Zn-SOD*, and *Histone H3* have been validated in somatic embryogenesis of longan (Lin and Lai, 2010); *RPL* has been studied in banana (Chen et al., 2011). *ACO* as the target gene was valuated to validate our results. Agarose gel electrophoresis, amplicon sequencing and dissociation curve analysis were used to verify the specificity of the RT-qPCR primer pairs (Figure S1). Standard curves were generated to calculate PCR amplification efficiency.

The appropriate reference gene should exhibit stable expression in different experimental conditions and in all samples (Chen et al., 2011; Zhong et al., 2011). However, no reference gene was found to have invariant expression in our experiments (Figure 1), which is consistent with the results obtained by Anna et al. (2009) and Jain et al. (2006), both of whom highlighted the importance of determining the appropriate reference genes under given conditions. In previous studies, the Cq values and mean Cq values ranged from 11 to 35 and 16 to 33, respectively, across all tested samples (Jain et al., 2006). In the current study, the Cq values ranged from 11.25 (*18SrRNA*) to 34.72 (*Cu/Zn-SOD*), and the mean Cq values lied between 17.43 (*18SrRNA*) and 29.02 (*Fe-SOD*). Variation (Cq_max_–Cq_min_) within an experiment was always less than 3, but the variation values across the tested samples were within the range of 8–15 (Table S1), which were higher than those reported by Zhong et al. (2011) and Chen et al. (2011) for all tested samples. The differences between the studies may be explained by use of different species and treatment conditions. Furthermore, these results showed it was urgently needed to compare the stability of the reference gene before using them for normalization in RT-qPCR assays (Chandna et al., 2012; Marum et al., 2012).

There was only one reference gene for normalization in RT-qPCR analysis in longan fruit (Zhao et al., 2011; Chen et al., 2012). While, many reports indicated that more than one reference genes should be used for normalization in RT-qPCR analysis (Vandesompele et al., 2002; Die and Rowland, 2013). The geNorm result showed that two reference genes were sufficient to normalization in RT-qPCR analysis in different fruit developmental stages, NAA, 2,4-D, ethephon, bagging, temperature and girdling with defoliation treatments, except in different varieties and organs. Three genes were sufficient for normalizing gene expression in different varieties of samples. When 0.15 as the cut-off value, none of the gene selected was appropriate across different organ samples. However, this cut-off value can be adjusted depending on experimental conditions. Based on our findings and previous reports (Vandesompele et al., 2002), we conclude that at least three most stable reference genes are needed for normalization in RT-qPCR under different organ conditions.

Previous reports showed that different sets of samples have their own most suitable reference genes (Chen et al., 2011; Zhong et al., 2011), and the same results were obtained in the present study (Figure 3). For example, *Fe-SOD* and *Cu/Zn-SOD* were the most stable genes under different organs. *GAPDH* and *Mn-SOD* genes ranked higher in different varieties of longan aril and fruit abscission zone in girdling with defoliation treatment, whereas *Fe-SOD* and *GAPDH* genes did better than *GAPDH* and *Mn-SOD* genes in the pericarp of longan fruit in different varieties. *GAPDH* and *EF-1a* were the most stable genes under NAA, ethephon treatment and fruitlets in girdling with defoliation conditions. *GAPDH* and *RPL* were the most suitable reference genes in aril samples at different developmental stages. *GAPDH* gene was one of the best reference genes in the seven experiments in this study, and the same result was found in litchi (Zhong et al., 2011), pear (Wu et al., 2012), plum (Kim et al., 2015), and Chinese wolfberry (Wang et al., 2013). Interestingly, *GAPDH* is one of the least stable genes in watermelon (Kong et al., 2014).

*Mn-SOD* and *EF-1a* genes were ranked higher in pericarp at different developmental stages. *Fe-SOD* and *EF-1a*, or *18SrRNA*, and *EF-1a* were the most suitable reference genes under bagging or cold temperature (4°C) treatment. *EF-1a* was one of the most suitable reference genes across six experiments, which is consistent with previous findings in litchi (Zhong et al., 2011), pear (Wu et al., 2012), berry (Reid et al., 2006), plum (Kim et al., 2015), rice (Jain et al., 2006), and *Lolium temulentum* (Dombrowski and Martin, 2009). However, *EF-1a* is one of the least stable genes in banana (Chen et al., 2011).

*18SrRNA* and *Actin* were the most stable genes in room temperature (22°C) treatment. *Actin* has been used as reference gene for normalization in many RT-qPCR studies (Kandasamy and Meagher, 1999; Reid et al., 2006), including those on longan fruit (Zhao et al., 2011; Chen et al., 2012). The present study showed that *Actin* was one of the most suitable reference genes but only at room temperature (22°C) and not in most other conditions. Therefore, more suitable reference genes should be considered in RT-qPCR analysis in longan fruit in future studies. Furthermore, previous studies demonstrated that *Actin* transcription in tissues subjected to cold treatment is less stable (Lopez-Pardo et al., 2012; Saha and Vandemark, 2012), suggesting that low temperatures influence its transcription and/or turnover rates. Consistent with this, *Actin* was previously reported as one of the least stable genes by Tatiane et al. (2015) and Wu et al. (2012). The *18SrRNA* was not the stable reference gene in Libault et al. (2008) report. In this research, *18SrRNA* was one of the most suitable reference genes under room temperature (22°C) treatment; however, in most other conditions, *18SrRNA* was either one of the most or least stable genes. Previous studies have also reported that this gene is unstable in peach (Tong et al., 2009), pear (Wu et al., 2012), melon (Carmen et al., 2015), zucchini (Obrero et al., 2011), and cucumber (Wan et al., 2010). In addition, *18SrRNA* gene expression was dramatically higher than those of other reference genes (Table S1), so *18SrRNA* is not an appropriate reference gene for normalization in RT-qPCR studies involving target genes with mid- or low-level expression.

*TUA* and *Histone H3* were the suitable reference genes under 2,4-D treatment. *Histone H3* was found to be one of the best reference genes in the 2,4-D treatment group, but it did not rank high in other treatment conditions. Notably, this gene was previously reported to be the least stable reference gene in citrus (Mafra et al., 2012). Interestingly, while *TUA* was found to be one of the most suitable reference genes under 2,4-D treatment, it was one of the least stable reference genes in most samples used in this study. Consistent with this, *TUA* was previously reported to be the least stable gene in litchi (Zhong et al., 2011), pear (Xu et al., 2015), zucchini (Obrero et al., 2011), and cucumber (Wan et al., 2010). Thus, a gene that is invariant in one experimental condition may have varying expression in other experiments. Therefore, verifying the stability of reference genes for each experimental condition is critical before using them for normalization in RT-qPCR assays.

The stability of the candidate reference genes was evaluated using geNorm and NormFinder algorithms in this study. While some studies have reported small differences in gene stability ranking generated by the two algorithms (Cruz et al., 2009; Lee et al., 2010), some have found large differences (Anna et al., 2009; Lin and Lai, 2010). In this study, slight differences were found between the geNorm and NormFinder results. The six most stable reference genes (half of the total) were similar, but the ranking order was different in the two algorithms (Figure 4 and Table S5). Moreover, the two most unstable reference genes identified by the two programs were the same (Table 3). These results are consistent with those reported by other groups (Marino et al., 2008; Artico et al., 2010; Lin and Lai, 2010; Wan et al., 2010). Given the differences in statistical algorithm used by geNorm and NormFinder programs, it is not surprising that there are some differences in their results. Nevertheless, there is substantial overlap in the results generated by the two programs, so use of both methods allows affirmation of candidate reference genes.

In orded to validate our results, relative mRNA levels of *DlACO* in the fruit abscission zone after girdling with defoliation treatment were calculated using different reference genes. Results showed that when used the most unstable reference gene result in overestimation expression (Figure 5), which indicated that an appropriate reference gene was urgently needed in RT-qPCR analysis.

## Conclusion

As far as we know, this study was the first to systematically select appropriate reference genes for RT-qPCR in preharvest and postharvest longan fruits. Accurate results could be obtained with the use of two or three appropriate reference genes for normalization by RT-qPCR in preharvest and postharvest longan fruits. The most suitable combinations used to normalize the expression profiles in preharvest longan fruit are as follows: *GAPDH* + *EF-1a* for NAA, ethephon treatment and fruitlets in girdling with defoliation conditions; *GAPDH* + *Mn-SOD* for fruit abscission zone in girdling with defoliation conditions; *GAPDH* + *RPL* or *Mn-SOD* + *EF-1a* for aril or pericarp at different developmental stages, respectively; *Fe-SOD* + *EF-1a* for bagging treatment; *TUA* + *Histone H3* for 2,4-D treatment; and *Fe-SOD* + *Cu/Zn-SOD* + *Mn-SOD* for different organs. In postharvest longan fruit, *18SrRNA* + *EF-1a* and *18SrRNA* + *Actin* were the most suitable combinations for 4 and 22°C temperature conditions, respectively, while *Fe-SOD* + *GAPDH* + *Cu/Zn-SOD* and *GAPDH* + *Mn-SOD*+ *EF-1a* were suitable for pericarp and aril of longan fruit, respectively. Analysis of *DlACO* gene expression indicated that use of suitable reference genes was crucial for getting accurate results in RT-qPCR analysis. Taken together, results from the present work provide a reference for choosing suitable reference genes for more accurate RT-qPCR analysis in preharvest and postharvest longan fruits.

## Author contributions

JW and HZ conceived and designed the experiments. YW and HZ performed the experiments. WL analyzed the data. SS and LL contributed reagents/materials. JW and YW wrote the paper. All authors read and approved the manuscript.

### Conflict of interest statement

The authors declare that the research was conducted in the absence of any commercial or financial relationships that could be construed as a potential conflict of interest.

## Supplementary material

Figure S1 **Single-peaked melt curves for all reference genes from three technical replicates of one set of cDNA pools with non-template control**.

Table S1 **Cq values of all samples in longan**.

Table S2 **Data statistics of Cq values of candidate reference genes in longan**. Mean, median, minimum, and maximun of Cq values were determined by statistical analysis.

Table S3 **Candidate genes ranked according to their expression stability values (M) estimated using the geNorm algorithm**.

Table S4 **Ranking of the candidate reference genes in different samples according to their stability value calculated by NormFinder analysis**.

Table S5 **Comparison of the ranking of candidate reference genes according to their stability value calculated by geNorm and NormFinder analysis**.

## Abbreviations

*ACO*: 1-aminocyclopropane-1-carboxylate oxidase
*Actin*: Actin
*Cu/Zn-SOD*: Copper/zinc-superoxide dismutase
*CYP*: Cyclophilin
*EF-1a*: Elongation factor 1-alpha
*Fe-SOD*: Iron-superoxide dismutase
*GAPDH*: Glyceraldehyde-3- phosphate dehydrogenase
*Histone H3*: Histone H3
*Mn-SOD*: Manganese superoxide dismutase
NAA: Naphthaleneacidic acid
PCR: Polymerase chain reaction
*RPL*: Ribosomal protein L
RT-qPCR: Reverse transcription quantitative PCR
*TUA*: Alpha-tubulin
*TUB*: Beta-tubulin
*UBQ*: Ubiquitin
*18SrRNA*: 18S ribosomal RNA
2,4-D: 2,4-(dichlorophenoxy)acetic acid.
