# Supplementary figures and images for: Validation of Reference Genes for RT-qPCR Studies of Gene Expression in Preharvest and Postharvest Longan Fruits under Different Experimental Conditions
Source: Front Plant Sci. 2016 Jun 3;7:780. doi: 10.3389/fpls.2016.00780 (PMC4891570; doi:10.3389/fpls.2016.00780)

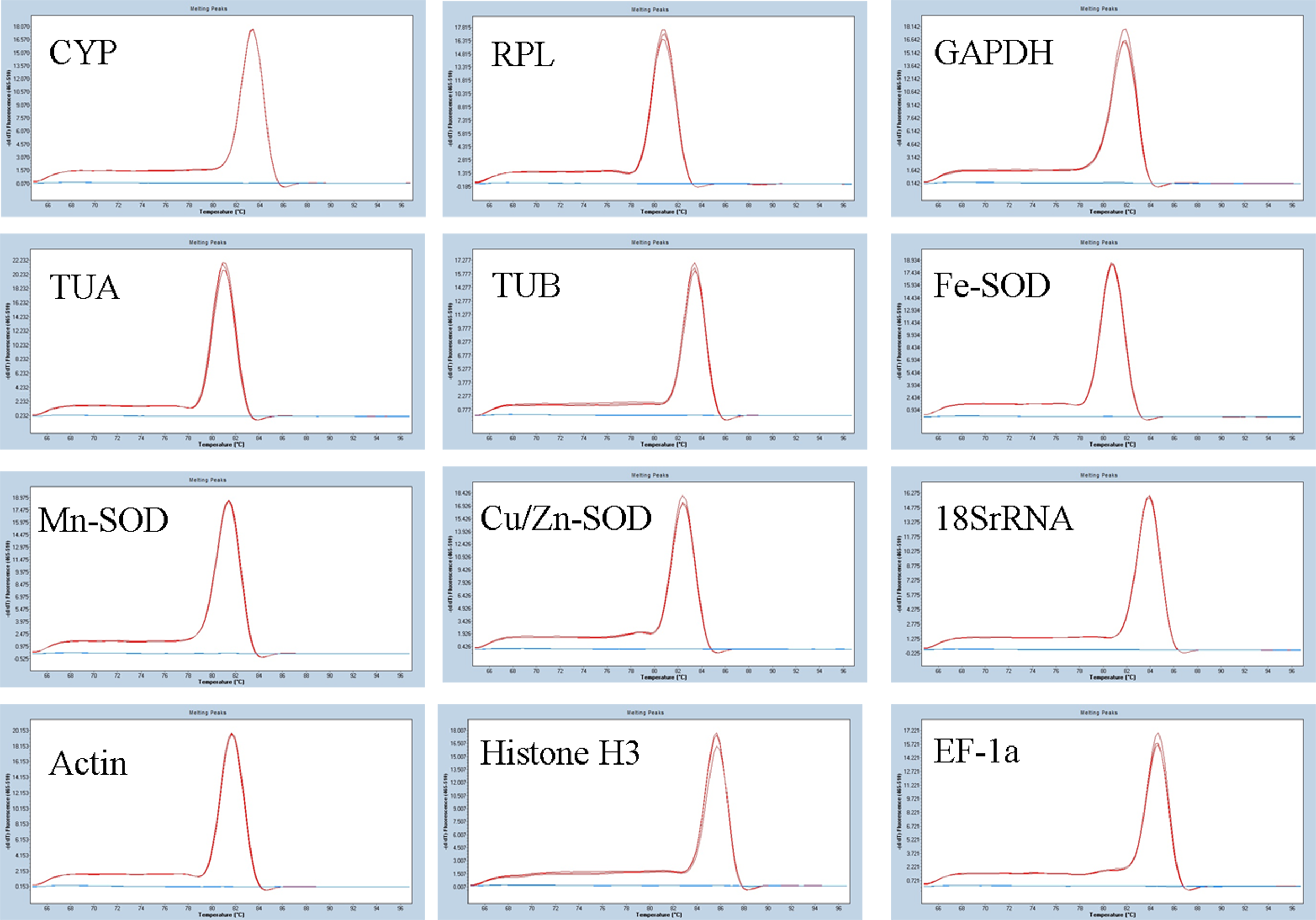

Supplement: Figure S1 — Single-peaked melt curves for all reference genes from three technical replicates of one set of cDNA pools with non-template control. [file Image1.TIF]
